# *LPIN1* rhabdomyolysis: A single site cohort description and treatment recommendations

**DOI:** 10.1016/j.ymgmr.2022.100844

**Published:** 2022-02-05

**Authors:** Navya Kanderi, Brian Kirmse, Debra S. Regier, Kimberly A. Chapman

**Affiliations:** a Children's National Rare Disease Institute, Washington, DC, United States of America; b Howard University Medical School, Washington, DC, United States of America; c University of Mississippi, Jackson, MS, United States of America

**Keywords:** LPIN1, Rhabdomyolysis

## Abstract

Individuals with *LPIN1* deficiency have early recurrent, life-threatening rhabdomyolysis but the full phenotypic spectrum and optimal treatment of the disorder remains unknown. Here we report the clinical details and treatment outcomes of 6 patients from our health system. The average age of presentation in our cohort was 23.8 months ±11.6 months (range 15–46 months). The average number of days for each hospitalization for this cohort is 11.7±13.2 days. Creatinine kinase (CK) levels peak during our care averaged 607,725 units/L (range 157,000-1,100,000 units/L). We observed that aspartate aminotransferase levels paralleled the CK levels in its elevation and resolution (Pearson's correlation *R* = 0.995); while alanine aminotransferase paralleled the elevation but lagged in the resolution of CK levels (*R* = 0.728). Unlike historical accounts, in our patient population, rhabdomyolysis was sometimes seen without inciting viral or traumatic events. We also cared for multiple individuals that had received treatment at other centers. This allowed us to compare multiple practice approaches and led to a standardized Care Recommendations.

## 1 Introduction

Acute recurrent myoglobinuria (OMIM #268200) has recently been attributed to bi-allelic mutations in *LPIN1* [[1], [2], [3]]. The spectrum of clinical features includes myoglobinuria, myalgia, hypotonia, muscle weakness, decreased reflexes, and in some severe cases, acute renal failure. Rhabdomyolysis is a condition in which injured skeletal muscle breaks down, leading to a release of the muscle fiber contents into the blood, which can be clinically monitored by measuring creatine kinase levels (CK) [2,3]. While rhabdomyolytic episodes can occur without a significant trigger, they often present after febrile illness, strenuous exercise, and fasting [2,4]. Multiple cases have been reported of sudden death from arrhythmia, hypothesized to be due to hyperkalemia [[5], [6], [7]]. The underlying genetic etiology of rhabdomyolysis is expanding as molecular testing has led to classifications of various conditions. *LPIN1* deficiency is diagnosed through clinical evaluation, detailed history, laboratory studies evaluating CK and urine myoglobin levels, muscle biopsy (no longer recommended), and genetic testing to distinguish inherited from acquired causes of rhabdomyolysis.

*LPIN1* deficiency is an autosomal recessive disease [1].The pathophysiological basis of *LPIN1* deficiency, including the associated rhabdomyolysis, has not be fully elucidated. Lutkewitte and Finck recently proposed that the ability of Lipin1, the protein product of LPIN1, to lead to regulation of the amounts of diacylglycerol and phosphatidic acid outside of adipose tissues impacts critical signaling cascades. This leads to alterations in cellular homeostasis in mammalian organisms (reviewed in Lutkewitte and Finck [8]). The *LPIN1* gene expresses a magnesium-ion-dependent phosphatidic acid phosphohydrolase enzyme that catalyzes the dephosphorylation of phosphatidic acid to yield diacylglycerol and is consequently required for adipocyte differentiation [1,6,9]. In mouse models, decreased gene expression leads to skeletal myopathy [10]. Jiang et al. showed that lipin1-deficient mice lack the ability to properly regenerate skeletal muscle, showing the importance of this lipid regulator on the overall stability of skeletal muscle [11]. The current working model of the mechanism of disease includes that loss of Lipin1 function impact normal phosphatidic acid metabolism. This leads to an alteration in the signaling pathways for fatty acid metabolism and signal transduction responses. For example, by disturbing its role in communications to various organelles during stress leading to unregulated stress response, abnormal function of mitochondria in terms of fusion and fission, altered fatty acid oxidation and impaired formation of these autolysosomes in muscle, resulting in rhabdomyolysis due to muscle cell death [9].

Here we report an additional 6 individuals with LPIN1 deficiency and their phenotype. In addition, since all the patients were evaluated at the same facility for at least one of their hospitalizations, we provide the quantitative values for their laboratory findings. Furthermore, we describe the effects of various treatments on time to recovery.

## 2 Case reports

All research has been performed in accordance with the Declaration of Helsinki and is approved by the Children's National Institutional Review Board (Pro0014817). The information reported was obtained via a retrospective review of medical records obtained from patients with *LPIN1* mutations who were seen in our clinic and/or hospital. Data were abstracted from our hospitals electronic medical record by KAC and de-identified.

This case report details six patients with *LPIN1* deficiency and their varying presentations including range in creatine kinase (CK levels) and potassium levels (K levels), varying symptom presentations, and therapeutic treatments. The case reports are as follows and it summarized in Table 1. None of our patients are from consanguineous parents and there is no family history unless noted in the reports below. All sequencing results were done in CLIA approved clinical laboratories. Reported genotypes correlate to the report provided by the testing laboratory.

**Table 1:** Six patients with their age of onset, Characteristics of the cohort of patients with lipin1 deficiency evaluated and treated at one institution. Abbreviations: D10: 10% dextrose solution, bicarb: Bicarbonate, CVVHD: continuous veno-venous hemodialysis, D7: 7% dextrose solution, URI: upper respiratory infection. Gender listed is gender assigned at birth.

	Age of onset	Presenting symptom	Admis-sions (#)	Average length of hospitalization (days)	CK (max)	AST/ALT units/L (max)	K (max)	Therapy used	Last follow-up age	Baseline CK levels (units/L)	Genetic change(s)
Patient 1 Female	2 years	lethargy, mental status changes during ear infection	3	16.7±14.2	1.1 million units/L	15,047/ 3921	8.3 mmol/L	D10 fluids, bicarb, sedation/intubation	7 years	200 s	c.1535 + 4_1535 + 7delAGTA (Novel) and Deletion of exons 20–21
Patient 2 (Male)	23 months	decreased activity, leg pain	3	8.8 ±8	394,375 units/L	10,600/ 3348	5.7 mmol/L	D10 fluids CVVHD	6 years	120 s	Homozygous deletion of at least exon 2
Patient 3 (Male)	4 years	pain and refusal to walk	4	5.3±3.2	151,770 units/L	649/626*	5.4 mmol/L	D10 fluids, bicarb.	14 years	200 s	homozygous deletion exon 18–19 [2.3]
Patient 4 (Male)	14 months	pain and refusal to walk	2	6	131,200 unit/L	ND	4.6 mmol/L	D10 fluids, bicarb	6 years	140 s	0.5 kb deletion in intron 19; no second found
Patient 5 (Male)	15 months	lethargy during bronch-iolitis	2	19±20	919,000 units/L	17,090/ 5400	8 mmol/L	D7–10 fluids CVVHD	12 years	150 s	c.2174G > A; p.R725H (Novel) and deletion 18–19 exon [2,3]
Patient 6 (Male)	2 years	muscle pain corresponding to URI	10	3.9 ±2	950,000+ units/L	325/ 85**	5.6 mmol/L	D10 fluids Insulin, solumedrol	6 years	150–200 s	C.825G > AP·W275* (Novel) and C.1699-2A > G (Novel)

*CK at that time was 16,190 units/L **max CK 29,625 units/L this hospitalization; ND not done or not in records.

### 2.1 Patient 1

**Fig. 1:**
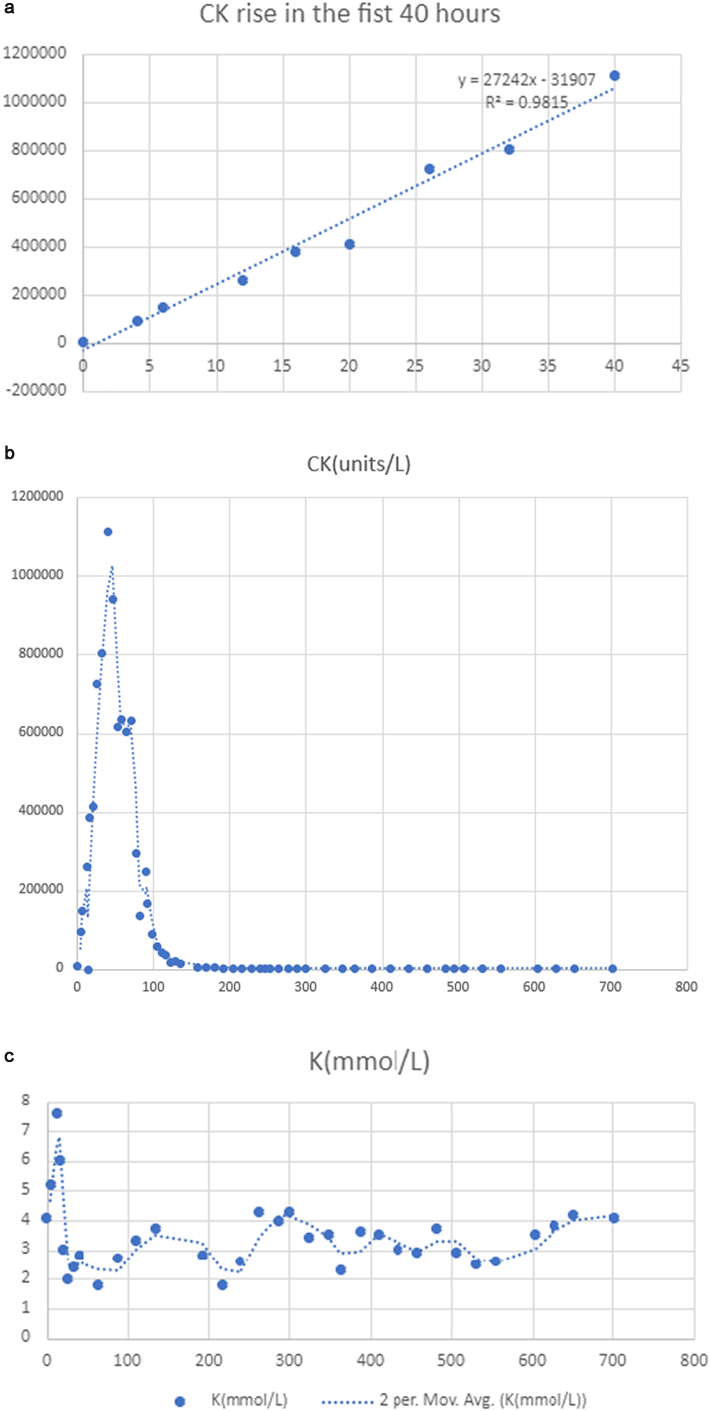
A. CK levels for the first 40 h of subject 1 episode #3 as it increases (Approximately 2500 units/L CK increase per hour during the first 40 h). B. CK (units/L measures for the entire 700 min for event #3, subject 1 as demonstrated in Table 2. C. potassium levels (mmol/L) for 700 h.

Patient 1 presented at 2 years old with lethargy and mental status changes during an ear infection. Over 4 years, the patient had associated weakness and three admissions complicated by elevated creatinine kinase and potassium in which her CK levels were in the 200 units/L range when healthy and peaked at 1.1 million units/L during acute illness. During one episode, the maximum potassium level preceded maximal CK time frame and was 8.3 mmol/L (Table 2, Fig. 1C). CK level obtained before and after the K level were 262,100 and 386,300 units/L (Table 2, Fig. 1A and B. She was treated for the elevated K levels; thus, follow-up K levels were in response to treatment. Therapy during her most severe presentation was initiated with fluid resuscitation (10% dextrose, 77 mmol/L sodium chloride, 20 mEq/L sodium bicarbonate) at 1.5 times her maintenance rate. An insulin drip was used to maintain glucose levels of 80–250 mg/dL to avoid decreasing the total glucose infusion rate during treatment. During her most severe decompensation, fluid-only treatment failed. During that episode (Fig. 1, Table 2), patient 1 required sedation (within 24 h), paralysis (within 24 h), intubation (within 24 h), and was treated with dexamethasone (at about 40 h). We have the most early presentation laboratory data from this event and thus, using as an illustration. Clinical genetic testing demonstrated c.1535 + 4_1535+ 7delAGTA and deletion of exons 20–21. Age at last follow up was 7 years old. Family's reported ethnicity is Caucasian.

**Table 2:** Time course for increases (and decreases) of K levels (mmol/L), CK levels (units/L), AST (units/L) and AST (units/L) for Subject 1 (Episode #3) illustrates the rapid rise of CK and K level with peak at 40 h and 14 h respectively. Time 0 is defined as the first laboratory draw taken (in the emergency room). nd: not done (measured).

Time (hour)	Potassium (mmol/L)	CK (units/L)	AST (units/L)	ALT (units/L)
0	4.1	6851	196	35
4	5.2	96,800	1563	302
6	nd	149,800	nd	nd
12	7.6	262,100	3462	815
14	8.3	nd	nd	nd
16	6	386,300	4578	1171
20	3	413,920	4965	1119
26	2	727,375	9089	1960
32	2.4	806,000	12,443	2945
40	2.8	1,113,400	15,047	3826
46	2.7	942,400	nd	nd
52	nd	618,125	nd	nd
58	nd	635,000	nd	nd
64	1.8	604,625	8007	3921
70	nd	631,840	nd	nd
76	2.6	294,375	nd	nd
82	nd	135,120	nd	nd
89	2.7	247,625	3129	3620
92	nd	166,600	nd	nd
98	nd	89,250	nd	nd
104	nd	57,250	nd	nd
110	3.3	42,125	1151	3158
116	nd	36,208	nd	nd
122	nd	16,649	nd	nd
128	nd	19,600	nd	nd
135	3.7	14,880	591	2688
157	nd	6197	nd	nd
168	3.3	4367	nd	nd
180	3.7	3920	nd	nd
192	2.8	3285	141	1133
204	nd	2432	nd	nd
216	1.8	1730	78	696
228	nd	1540	nd	nd
240	2.6	1462	61	462
246	nd	1232	nd	nd
252	3.3	1410	nd	nd
264	4.3	1044	68	380
276	nd	1189	nd	nd
288	4	1003	65	325
300	4.3	743	68	278
325	3.4	568	39	201
348	3.5	355	30	136
363	2.3	276	20	83
387	3.6	345	23	81
411	3.5	298	20	66
435	3	239	17	55
459	2.9	229	20	51
483	3.7	277	21	43
495	nd	419	nd	nd
507	2.9	434	31	38
531	2.5	431	27	34
556	2.6	440	28	37
604	3.5	583	31	34
628	3.8	651	38	35
652	4.2	723	38	37
702	4.1	645	39	35

### 2.2 Patient 2

Patient 2 was 23 months old at the first onset of symptoms. The patient presented with decreased activity and leg pain. During rhabdomyolysis crisis, the peak CK levels was 394,375 units/L. Continuous veno-venous hemodialysis was initiated for therapy due to the severities of the symptoms and lack of response to fluid treatment. Genetic testing showed a homozygous deletion of exon 2. Age at last follow up was 6 years old with baseline CK levels of 120 units/L. The patient's family history is notable for a sibling with sudden death after presenting with similar complaints. Cause of sibling's death was hypothesized to be due to arrhythmia based on medical record reviews. CK levels were not obtained at the time of the sibling's death. Family's reported ethnicity is Caucasian, Scandinavian, and English.

### 2.3 Patient 3

Patient 3 first presented at age 4 with pain and refusal to walk. Other associated symptoms the patient had were abdominal, arm and leg pain. Baseline CK levels were in the 200 s units/L with CK level peaking at 151,770 units/L during a rhabdomyolysis decompensation event. This patient responded to fluid-only treatment (10% dextrose with 77 mmol/L sodium chloride at 1.5 times maintenance rate). Follow up genetic testing showed homozygous deletion exon 18–19 (initially reported in [2] and [3]). Age at last follow up was 14 years old. Family's reported ethnicity is German, Welsh, English, and Native American.

### 2.4 Patient 4

Patient 4 presented to our institution with pain and refusal to walk at 3 years old. He had been admitted with a previous episode of rhabdomyolysis prior to his diagnosis at 15 months of age to a different institution. The patients' baseline CK levels range in the 140 s units/L but peaked at 131,200 units/L with decompensation during his 3-year-old presentation. Peak K level was recorded at 4.6 mmol/L. He responded to fluid therapy (10% dextrose with 77 mmol/L sodium chloride at 1.5 times maintenance rate). Genetic testing demonstrated 0.5 kb deletion in intron 19. Age at last follow up was 6 years old. Family's reported ethnicity is Salvadorian.

### 2.5 Patient 5

Patient 5 initially presented with lethargy, bronchiolitis, and weakness at 15 months of age. His baseline CK was 150–160 units/L when well and the highest recorded CK level was 919,000 units/L. Their highest K level was 8 mmol/L. During his most severe episode, he failed fluid therapy and required veno-venous hemodialysis. Genetic testing showed heterozygous changes with c.2174G > A; p.R725H (novel) and deletion at exon 18–19 (previously reported [2] and [3]). Age at last follow up was 12 years. Family's reported ethnicities include Irish, German, Scottish and Native American.

### 2.6 Patient 6

Patient 6 first presented with symptoms of pain in the legs and rhabdomyolysis at 2 years of age triggered by an upper respiratory infection. Past history included multiple admissions for rhabdomyolysis due to decompensations. Peak CK level for this patient was recorded as 950,000+ units/L (exceeded maximum value available where testing performed) and highest K level was 5.6 mmol/L. During this event, they responded to fluid therapy (10% dextrose with 154 mmol/L sodium chloride delivered at 2-times maintenance rate) and required an insulin drip to maintain euglycemia. Due to severity of the presentation, steroids were administered (solumedrol 0.6 mg/kg/day). Genetic mutation demonstrated C.825G > A; p.W275* (novel) and C.1699-2A > G (novel). Age at last follow up was 6 years old. Family's reported ethnicity is mixed European, Puerto Rican and Panamanian.

## 3 Results

The average age of presentation was 23.8 months ±11.6 months (range 15–46 months). Based on the reported ages of individuals who presented in the literature (see Table 3), 38 +/− 24 months (range 3 months to 7 years), our cohort was identified earlier in life. The average number of days for each hospitalization for this cohort is 11.7±13.2 days. We intubated and sedated two of these individuals due to the severity of their symptoms. As expected for severity, individuals requiring intubation and sedation for management had the longest hospital courses.

**Table 3:** Reports from the literature for individuals with *LPIN1* deficiency Twenty-four individuals have been previously described with lipin-1 rhabdomyolysis. Patients 1, 2 [5]; Patients 3, 4, 5 [2]; patient 6 [6]; patient 7 [20]; patient 8 [4]; patient 9 [21]; patients 10–18 [7]; patient 19–22 [7], Patient 23 [10], Patient 24 [22]. Abbreviations: NS: normal saline, D dextrose, IVF: Intravenous fluids, Y year: amp: Amplitude, CHO: carbohydrate, COQ10: Coenzyme Q10, FHX; family history, CRRT: continuous renal replacement therapy.

	Presentation & history	Age (present-ation)	Baseline CK	Max CK (units/L)	Alive or deceased	Intervention
Patient 1	Cardiac arrest; symmetric high amp T waves	6 y		22,013	Deceased	3 L/m^2^/d NS + D
Patient 2	Muscle paint, brown urine, widening QRS followed by arrest; Diffused symmetrical high Amp T waves, prolonged QRS	5 y		55,500-213,107	Deceased, Cardiac hypertrophy consistent with chronic hypertension	Hyperhydration NS + D
Patient 3	Recurrent myoglobinuria with febrile illness; intermittent stuporous	Around 2 y		180,000-450,000	Alive	IVF with alkalizations
Patient 4	Recurrent myoglobinuria with febrile illness	Around 2 y		180,000-450,000	Alive	IVF with alkalizations
Patient 5	Recurrent myoglobinuria with febrile illness	Around 7 y		180,000-450,000	Alive	IVF with alkalizations
Patient 6	At 22 m following respiratory infection. 25 m, acute muscle pain and weakness following fasting and strenuous exercise	22 m	500–2000 between episodes	250,000-500,000	Alive (FHX of 2 siblings who died at 2 and 4 years	Aggressive CHO, MCT oil, regular COQ10, high calorie drink prior to Physical activity (limit exercise to 20 min); dexamethasone
Patient 7	Multiple presentation of rhabdomyolysis associated with mild febrile illnesses or decreased calories, including follow surgery.	<7 y	baseline 250–300	At least 2 episodes CK >180,000		D10NS, avoid propofol and Suxamethonium, continued after
Patient 8	6 rhabdomyolysis episodes, 3 when febrile	4 y			Alive	Hydration, calories, electrolyte replacement, carnitine. Episodes 4–6 dexamethasone
Patient 9	9 y “cola-colored urine”, exercise and fasting about 12 h, admitted to ICU	19 m			Alive	CRRT
Patient 10	Exercise intolerant, renal dysfunction, myoglobinuria			37,787	Alive	
Patient 11	Exercise intolerance, renal dysfunction during			15,000	Alive	
Patient 12	Myoglobinuria with febrile illness			296,000	Alive	
Patient 13	Myoglobinuria			36,000	Alive	
Patient 14	Myoglobinuria			142,000	Deceased	
Patient 15	Myoglobinuria	16 m		32,668	Alive	
Patient 16	Myoglobinuria	2 y		70,000	Alive	
Patient 17	Myoglobinuria	8 m		200,00	Alive	
Patient 18	Normal or slightly elevated CK without signs of muscle weakness	2.5 y		500,000	Deceased	
Patient 19	Normal or slightly elevated CK without signs of muscle weakness	2.5 y		706,800	Alive	(Patents 19–22) Treated with hyperhydration (3 L/m^2^/day of a 10% glucose solution with 80 mmol/L sodium chloride and 20 mmol/L potassium chloride) and forced diuresis. A high-concentration glucose solution was given to establish anabolism as quickly as possible. When necessary, insulin therapy was started to control hyperglycemia
Patient 20	Normal or slightly elevated CK without signs of muscle weakness	6 y		140,610	Alive	
Patient 21	Normal or slightly elevated CK without signs of muscle weakness	4 y		625,000	Alive	
Patient 22	Normal or slightly elevated CK without signs of muscle weakness	5y		140,040	Alive	
Patient 23	Normal strength	16 m	164	498,800	Alive	
Patient 24		26 m		943,452	Alive	

CK levels in this cohort of LPIN1 patient increase at a faster rate than our cohort with LCHAD over the first several hours—especially in the first 40 h. In individuals that presented early with mild symptoms, the maximum CK level peaked at 3 h to 70 h following presentation. Tracking initial rise in CK levels can be difficult since not all patients in this cohort initially presented to our institution. However, patient 1's third admission is particularly informative since we have all her initial outside laboratory results as well as her hospitalization results (Table 2, Fig. 1A-C). In this case, the early presentation with refusal to walk led to immediate evaluation and admission and allowed for CK tracking with fluid management.

One patient had evidence of an arrhythmia. This occurred when they had a K level of 8.8 mmol/L, no concurrent CK level was obtained, but the CK levels obtained immediately prior and following were 262,100 and 386,300 units/L. The peak K level preceded the CK peak..

**Fig. 2:**
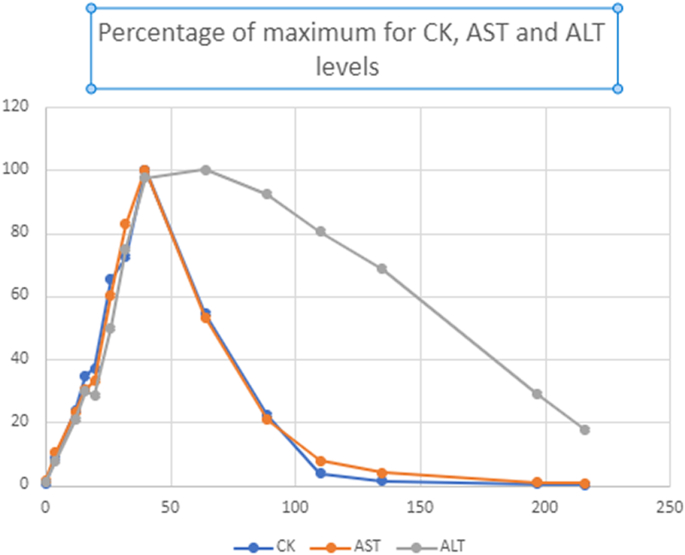
Percentage of maximum levels for CK, AST and ALT during the first 216 h of a rhabdomyolysis decompensation in Patient 1, episode 3 (non-normalized levels Table 2). As measured as the percentage of the maximum level detected during an episode, AST (Orange) and CK levels (Blue) correlate in the rise and fall (Pearson's correlation *R* = 0.995. ALT rises at similar rate, but it falls more slowly (Gray). X-axis is time (Hours), y-axis measures percentage of maximum level.

Early in this study, transaminase levels were evaluated on initial admission in this cohort, but frequently not thereafter. Based on the elevations and the pathophysiology of transaminase release from acutely injured muscles, we began to track these levels as a second marker for muscle breakdown. The transaminases are released by muscle as it breaks-down; thus, aspartate aminotransferase (AST) and alanine aminotransferase (ALT) appear to serve as secondary markers of muscle breakdown. In fact, the AST slope of rise and recovery paralleled the CK levels (Pearson's correlation *R* = 0.995)(and Fig. 2). The ALT rise parallels the CK levels; however, the drop in ALT levels lagged after the CK resolution. Thus, ALT elevations appear to be a longer-term marker of previous CK elevations (Fig. 2).

## 4 Discussion

Here, we report the clinical courses for an addition 6 new patients with LPIN1 deficiency and report and chart their CK levels, K levels, as well as AST/ALT levels. From these results and new knowledge about the pathophysiology of Lipin1 function, we have devised a set of guidelines from which we base our treatment for decompensations.

One important question is why do patients with *LPIN1* mutations, which impact the metabolism of de novo synthesis of glyceropholipids, develop rhabdomyolysis with illness? Recent studies in humans indicate that one contributory factor may be the tissue distribution of Lipin1. While there are multiple isoforms of Lipin, only expression of Lipin1 has been identified in human skeletal muscle [9]. De novo synthesis of glyceropholipds is highly regulated [9] and so coupled with the observation of accumulation of cholesteryl esters in muscle biopsies isolated from *LPIN 1*–deficient patients that these may indicate the cellular response to impaired triacylglycerol synthesis may include inflammation [9].

Lipin1 may also play a significant role in inducing gene expression related to modulation of inflammatory markers [12]. Thus, the idea of using steroids as a possible therapeutic approach is reasonable and logical. Previously, a cohort of individuals with non-Lpin1 caused rhabdomyolysis was described with a shortened time to CK levels resolution with steroid administration [13]. In the cohort described here, steroids were used with positive effects and this has been added to the recommendations for treatment for fluid-resistant rhabdomyolysis.

Lipin1 plays a role in regulation of fatty acid oxidations and mitochondrial function [14]. Thus, approaches to counteract the accumulation of fatty acid production during stress may be effective therapies [9] This has been demonstrated by showing accumulation of abnormal mitochondria and the presence of autophagosome structures in muscle biopsies. This observation is consistent with a defect in autophagic clearance given LPIN1 role in mitochondrial fission and fusion [14]. Based on these observations, the cohort described was treated with a glucose infusion rate of 6–10 mg/kg/min to prevent lipid breakdown and to increase support for stressed mitochondria.

This study was limited by the data available for each patient. For safety reasons, patients with lipin1 deficiency are directed to the closest hospital available if there are any concerns for a rhabdomyolysis event. Many institutions do not provide exact levels of CK after a certain level (i.e., greater than 95,000 units/L). Thus, we have chosen to also track a second marker, AST, since this enzyme was shown to parallel the CK levels during rhabdomyolysis events at our center, where the full range of CK levels were monitored. Thus, high levels of AST would alert the clinician to the risk for rhabdomyolysis and can serve as a secondary when true CK levels cannot be obtained. Moreover, the delayed normalization of the ALT level was helpful in predicting the trajectory for patients. For example, by following the ALT levels we could best assess if the CK values were up or down trending from a peak value (Table 2).

It is important to note that the rise in CK level is extremely fast in this patient population. The average rise for our cohort of CK was 2800 units/h, in the first 24 h despite initiation of fluid management. In comparison, the rise seen in one of our severe long chain hydroxy acyl dehydrogenase patients during a rhabdomyolysis event was 200–300 units/h in the first 24 h of illness (Chapman et al. Unpublished data).

Phenotype/genotype correlation has not been possible in the small cohort to date. Severe phenotypes were observed in those with missense and exonic deletions. We also showed that the age of our cohort is younger and maximum CK levels higher than those previously published. It is unclear if this was due to the bias of this center to assess CK levels in very ill children, based on experience, or some type of cohort bias.

Unlike previous publications, in our cohort, patients have episodes of rhabdomyolysis without inciting factors. We hypothesize that this might be due to mild viral or bacterial illnesses that occur in young children without symptoms that are clinically noted. This led to high use of CK evaluations for even the mildest of symptoms in this younger, more severe cohort. And likely, we may have identified individuals that would have previously had potentially life-threatening cardiac arrhythmias early. Historically, *LPIN1* mutation induced rhabdomyolysis was hypothesized to be caused by a combination of genetic susceptibility and an environmental risk [15]. In this case series, lack of known inciting environmental factors increases the risk for severe disease based on genetic susceptibility independent of environmental factors.

**Fig. 3:**
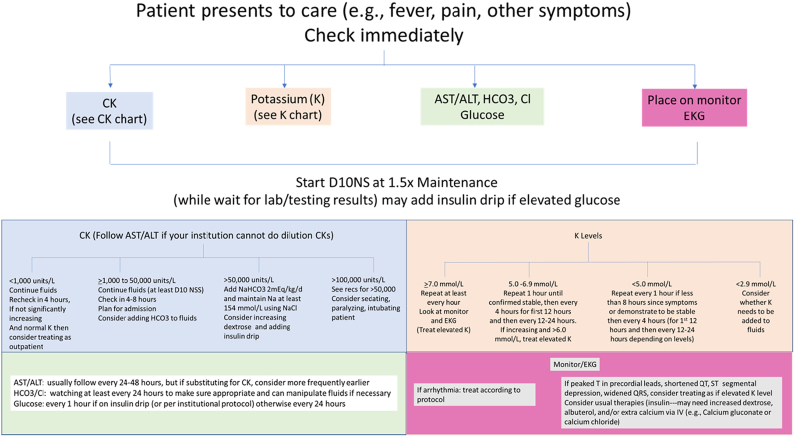
Summary of the protocol for individuals with *LPIN1* deficiency.

Based on the historical data and the six patients presented here, we have developed a protocol to treat those suspected or known to have *LPIN1* deficiency. This protocol prioritizes 1) providing adequate hydration and calories, AND 2) measure life threatening complications (e.g., rapid increase in K levels) (Fig. 3). For each patient who presents emergently with pain (or in younger patients, refusal to move), an intravenous catheter is placed urgently and 10% dextrose with 0.9% (154 mmol/L) sodium chloride at 1.5× maintenance rate (providing about 6–10 mg/kg/min glucose infusion rate depending on the patient's weight) is started. Our cohort received 7–10 mg dextrose /kg/min due to use of 10% dextrose and their weights based on using 1.5× maintenance calculations. This glucose infusion rate has been historically chosen to slow catabolic demand, based on data showing that a dextrose delivery rate of 6–10 mg/kg/min was shown to be adequate to replace gluconeogenesis in patients with glycogen storage disease, type 1 [16].

If low bicarbonate (<20 mmol/L) or CK greater than 50,000, sodium bicarbonate is added to deliver at least 2 mEq/kg/day and mixed with sodium chloride for a sodium goal (total of 154 mmol/L). The addition of sodium bicarbonate allows more balanced fluids; decreased risk for hyperchloremia; and alkalization of the urine, which has a historical role in renal protection [17]. Dialysis using can be added for extreme elevation of potassium, other severe electrolyte imbalances, fluid overload, renal dysfunction.

Potassium (K level) is monitored every 1–12 h (depending on timing in relation to first symptoms and initial level at presentation) (Fig. 3). If the initial K level is elevated and/or patient is early in their course (<8 h since initial pain symptoms), K level is monitored every 1–2 h to assess the rate of rise. In our cohort and others with LPIN1, the most rapid rise in K levels occurs in the first 8 h of symptoms and so particular attention is paid during this time.

Extremely elevated K levels (>7 mmol/L) are rare in our cohort. Due to reports of arrhythmias and family history of a sudden death in a child concerning for a fatal arrhythmia, all known patients are placed on a cardiac monitor. Special attention is paid to early signs of elevated K levels including widening of the QRS complex on EKG/monitor, peaked T waves in precordial leads, shortened QT interval, and ST segment depression.

If severe hypokalemia (<3.0 mmol/L) develops, which often occurs later in the treatment course due to fluids without potassium being used, replace potassium, as necessary. Our data indicate that low K levels are not usually observed before 24 h from initial symptoms, especially if given fluids without potassium.

Many individuals will require insulin to maintain normoglycemia with this protocol. This is physiologically important since hyperglycemia is evidence of poor glucose uptake by stressed muscles. Thus, insulin should be considered before reducing the glucose infusion rate of the fluids. The use of insulin should also lead to careful K level monitoring since insulin can lead to iatrogenic hypokalemia. As implied above, early initiation of an insulin drip allows for improved glycemic control as well as a transition away from fatty acid oxidation towards glycolysis.

Phosphorus and magnesium should be monitored every 6–12 h and replaced as is necessary.

We do not routinely check or follow urine myoglobin since levels would not alter our therapeutic interventions.

If CK levels are particularly elevated (>100,000 units/L) or rapidly rising without a treatable underlying cause, additional intervention should be considered. To prevent increased catabolism and stress, sedation, paralysis, and intubation should be considered. This also permits the use of higher doses of opioid pain medications, which would otherwise be avoided due to suppression of the respiratory state. We frequently check CK levels every 4–8 h until it peaks. We grant the half-like for CK is about 24 h, but using CK levels to create a timeline of illness helps with clinical decision making and decisions concerning escalation of care. As we de-escalate care, the frequency of CK levels checks becomes closer to every 24 h.

Finally, recent studies by Pichler et al. and Summerlin et al. has led to our recent addition of dexamethasone (0.6 mg/kg max day) in patients with fluid-unresponsive rhabdomyolysis, a history of difficult hospital course requiring management beyond fluids alone, or a history of arrythmia [13,18]. While our small cohort has not allowed a large enough study group to prove the benefit of steroid treatment, we do recommend this be considered in cases of *LPIN1* deficiency with complex or prolonged rhabdomyolysis events.

In summation, the cohort described and those in the literature indicate that the rhabdomyolysis in patients with *LPIN1* deficiency can be seen in a setting of an inciting factor or when one is not identified; should be monitored by use of either dilutional CK levels or co-monitoring of transaminase levels; treated with high glucose fluids with or without bicarbonate supplement based on laboratory values; monitored for elevated K levels [19] that may lead to arrythmias; and have anti-inflammatory treatment (i.e., dexamethasone) considered for severe case presentations.

## Declaration of Competing Interest

None.

## References

[1] Reue K. (2009). The lipin family: mutations and metabolism. Curr. Opin. Lipidol..

[2] Zeharia A., Shaag A., Houtkooper R.H., Hindi T., de Lonlay P., Erez G., Hubert L., Saada A., de Keyzer Y., Eshel G., Vaz F.M., Pines O., Elpeleg O. (2008). Mutations in LPIN1 cause recurrent acute myoglobinuria in childhood. Am. J. Human Genet..

[3] Michot C., Hubert L., Brivet M., De Meirleir L., Valayannopoulos V., Müller-Felber W., Venkateswaran R., Ogier H., Desguerre I., Altuzarra C., Thompson E., Smitka M., Huebner A., Husson M., Horvath R., Chinnery P., Vaz F.M., Munnich A., Elpeleg O., Delahodde A., de Keyzer Y., de Lonlay P. (2010). LPIN1 gene mutations: a major cause of severe rhabdomyolysis in early childhood. Hum. Mutat..

[4] Meijer I.A., Sasarman F., Maftei C., Rossignol E., Vanasse M., Major P., Mitchell G.A., Brunel-Guitton C. (2015). LPIN1 deficiency with severe recurrent rhabdomyolysis and persistent elevation of creatine kinase levels due to chromosome 2 maternal isodisomy. Mol. Genet. Metabol. Rep..

[5] Bergounioux J., Brassier A., Rambaud C., Bustarret O., Michot C., Hubert L., Arnoux J.B., Laquerriere A., Bekri S., Galene-Gromez S., Bonnet D., Hubert P., de Lonlay P. (2012). Fatal rhabdomyolysis in 2 children with LPIN1 mutations. J. Pediatr..

[6] Stepien K.M., Schmidt W.M., Bittner R.E., O'Toole O., McNamara B., Treacy E.P. (2019). Long-term outcomes in a 25-year-old female affected with lipin-1 deficiency. JIMD Rep..

[7] Jaradat S.A., Amayreh W., Al-Qa'qa K., Krayyem J. (2016). Molecular analysis of LPIN1 in Jordanian patients with rhabdomyolysis. Meta Gene.

[8] Lutkewitte A.J., Finck B.N. (2020). Regulation of signaling and metabolism by lipin-mediated phosphatidic acid phosphohydrolase activity. Biomolecules.

[9] Zhang P., Reue K. (2017). Lipin proteins and glycerolipid metabolism: roles at the ER membrane and beyond. Biochim. Biophys. Acta Biomembr..

[10] Schweitzer G.G., Collier S.L., Chen Z., Eaton J.M., Connolly A.M., Bucelli R.C., Pestronk A., Harris T.E., Finck B.N. (2015). Rhabdomyolysis-associated mutations in human LPIN1 lead to loss of phosphatidic acid phosphohydrolase activity. JIMD Rep..

[11] Jiang W., Zhu J., Zhuang X., Zhang X., Luo T., Esser K.A., Ren H. (2015). Lipin1 regulates skeletal muscle differentiation through extracellular signal-regulated kinase (ERK) activation and cyclin D complex-regulated cell cycle withdrawal. J. Biol. Chem..

[12] Kim H.B., Kumar A., Wang L., Liu G.H., Keller S.R., Lawrence J.C., Finck B.N., Harris T.E. (2010). Lipin 1 represses NFATc4 transcriptional activity in adipocytes to inhibit secretion of inflammatory factors. Mol. Cell. Biol..

[13] Summerlin M.L., Regier D.S., Fraser J.L., Chapman K.A., Kafashzadeh D., Billington C., Kisling M., Grochowsky A., Ah Mew N., Shur N. (2021). Use of dexamethasone in idiopathic, acute pediatric rhabdomyolysis. Am. J. Med. Genet. A.

[14] Zhang P., Verity M.A., Reue K. (2014). Lipin-1 regulates autophagy clearance and intersects with statin drug effects in skeletal muscle. Cell Metab..

[15] Indika N.L.R., Vidanapathirana D.M., Jasinge E., Waduge R., Shyamali N.L.A., Perera P.P.R. (2020). Lipin-1 deficiency-associated recurrent rhabdomyolysis and exercise-induced myalgia persisting into adulthood: a case report and review of literature. Case Rep. Med..

[16] Tsalikian E., Simmons P., Gerich J.E., Howard C., Haymond M.W. (1984). Glucose production and utilization in children with glycogen storage disease type I. Am. J. Physiol..

[17] Long B., Koyfman A., Gottlieb M. (2019). An evidence-based narrative review of the emergency department evaluation and management of rhabdomyolysis. Am. J. Emerg. Med..

[18] Pichler K., Scholl-Buergi S., Birnbacher R., Freilinger M., Straub S., Brunner J., Zschocke J., Bittner R.E., Karall D. (2015). A novel therapeutic approach for LPIN1 mutation-associated rhabdomyolysis. Austr. Exp. Muscle Nerve.

[19] Palmer B.F., Carrero J.J., Clegg D.J., Colbert G.B., Emmett M., Fishbane S., Hain D.J., Lerma E., Onuigbo M., Rastogi A., Roger S.D., Spinowitz B.S., Weir M.R. (2021). Clinical management of hyperkalemia. Mayo Clin. Proc..

[20] Burstal R.J. (2018). Volatile anesthesia for a child with LPIN1 gene mutation and recurrent rhabdomyolysis. Paediatr. Anaesth..

[21] Che R., Wang C., Zheng B., Zhang X., Ding G., Zhao F., Jia Z., Zhang A., Huang S., Feng Q. (2020). A rare case of pediatric recurrent rhabdomyolysis with compound heterogenous variants in the LPIN1. BMC Pediatr..

[22] Topal S., Kose M.D., Agin H., Sari F., Colak M., Atakul G., Karaaslan U., Isguder R. (2020). A neglected cause of recurrent rhabdomyolysis, LPIN1 gene defect: a rare case from Turkey. Turk. J. Pediatr..

